# Involvement of exogenous polyamines enhances regeneration and *Agrobacterium*-mediated genetic transformation in half-seeds of soybean

**DOI:** 10.1007/s13205-016-0448-0

**Published:** 2016-06-28

**Authors:** Muthukrishnan Arun, Arunachalam Chinnathambi, Kondeti Subramanyam, Sivabalan Karthik, Ganeshan Sivanandhan, Jeevaraj Theboral, Sulaiman Ali Alharbi, Chang Kil Kim, Andy Ganapathi

**Affiliations:** 1 Department of Biotechnology and Genetic Engineering, School of Biotechnology, Bharathidasan University, Tiruchirappalli, Tamil Nadu 620024 India; 2 Department of Botany and Microbiology, College of Science, King Saud University, Riyadh, 11451 Kingdom of Saudi Arabia; 3 Department of Horticultural Sciences, Kyungpook National University, Daegu, 702-701 South Korea; 4 Laboratory of Biochemistry and Glycobiology, Department of Molecular Biotechnology, Ghent University, Coupure links 653, 9000 Ghent, Belgium; 5 Bharathiar University, Coimbatore, Tamil Nadu 641046 India

**Keywords:** Soybean, Half-seed, Spermidine, Spermine, Putrescine

## Abstract

The present work demonstrates the participation of polyamines (PAs) to improve direct regeneration and *Agrobacterium*-mediated transformation in soybean half-seeds. The inclusion of PAs to culture medium along with optimal plant growth regulators (PGRs) enhanced shoot induction [98.3 %; 4.44 µM N^6^-benzyladenine (BA) and 103.27 µM spermidine] and elongation [90.0 %; 1.45 µM gibberellic acid (GA_3_) and 49.42 µM spermine]. The polyamine putrescine (62.08 µM) alone greatly enriched root induction (96.3 %). The influence of PAs on transformed plant production was assessed by comparing optimized protocol (comprising PAs and PGRs) with a regeneration system involving only PGRs. Plant transformation was performed in half-seeds of cultivar DS 97–12 using strain EHA105 harboring pCAMBIA1301. Transgene expression and integration was confirmed by GUS staining, PCR, and Southern hybridization. The transformed explants/materials successively cultured on co-cultivation (BA and spermidine), shoot induction (BA and spermidine), shoot elongation (GA_3_ and spermine), and rooting medium (putrescine) showed enhanced transformation efficiency (29.3 %) compared with its counterparts (14.6 %) with respective PGR alone [BA, GA_3_, or indole-3-butyric acid (IBA)]. Overall findings of the study suggest that involvement of PAs improved T-DNA transfer during co-cultivation, and delivered most suitable condition for efficient regeneration/survival, which led to enhanced transformation efficiency in soybean.

**Electronic supplementary material:**

The online version of this article (doi:10.1007/s13205-016-0448-0) contains supplementary material, which is available to authorized users.

## Introduction

Soybean [*Glycine max* (L.) Merrill] is considered to be one of the most economically important crops because of its rich protein (40 %), oil (20 %), and carbohydrate (12 %) content. Soybean seeds also possess isoflavones, omega-3 fatty acids, phytic acid, tocopherols, phenolic compounds, saponins, and minerals. To date, many laboratories put a great deal of effort to improve soybean by genetic manipulation with value added agronomical and nutritional traits. Though tissue culture and genetic transformation techniques have been well established for agriculturally important dicotyledonous species, these techniques could not be efficiently applied for soybean (Ma and Wu 2008). For genetic improvement of soybean through biotechnological applications, an efficient plant regeneration protocol is a pre-requisite. Recently, half-seed explants of soybean are gaining importance toward in vitro regeneration (Radhakrishnan and Ranjitha kumari 2007; Ether et al. 2013; Janani and Ranjitha Kumari 2013) and *Agrobacterium*-mediated genetic transformation (Paz et al. 2006; Zia et al. 2010b). Half-seed explants possess advantages like availability within a short time (maximum 1 day) which reduces the total regeneration period, and the starting material for experiment can be obtained immediately on demand, since imbibed seeds are its source.

We previously demonstrated the positive influence and synergistic effect of polyamines (PAs) in combination with different plant growth regulators (PGRs) on shoot regeneration from cotyledonary nodes of soybean (Arun et al. 2014). The PAs are considered as a new class of PGRs and hormonal second messengers, and as one of the reserves of carbon and nitrogen at least in cultured tissues (Kakkar et al. 2000). The PAs are known to stimulate embryogenesis in *Panax ginseng* (Kevers et al. 2000), shoot regeneration in Chinese radish (Pua et al. 1996), Korean radish (Curtis et al. 2004), and *Capsicum frutescens* (Kumar et al. 2007). Even though the role of PAs on promoting plant morphogenesis has been reported in several crops, only a few attempts (in wheat, tobacco, and apricot) were made to study its involvement on improving *Agrobacterium*-mediated genetic transformation (Khanna and Daggard 2003; Kumar and Rajam 2005; Petri et al. 2005). Furthermore, to date, no report is available in soybean with respect to PAs on improving T-DNA delivery and transformation efficiency.

Increasing meristematic cells in explants, enhancing T-DNA delivery to target cells, improving explant regeneration frequency, and reducing difficulties on plant regeneration/survival by PAs could contribute to attain higher transformation efficiency in any genotype. Hence, the present investigation was designed to (1) optimize an efficient regeneration system for half-seeds of soybean using different PGRs and PAs, and (2) evaluate the influence of PAs on transformed plant production by comparing optimized protocol (comprising PAs and PGRs) with a regeneration system involving only PGRs.

## Materials and methods

### Cultivar and seed source

Indian soybean cultivar (cv.) DS 97–12 was used in the present study. The cv. DS 97–12 possess a determinate growth habit with light green leaves and white flowers. The plants are of non-shattering type and attain full maturity on 116 days. The cultivar shows significant resistant to yellow mosaic virus and is moderately resistant to stem fly. The seeds of cv. DS 97–12 were procured from Indian Agricultural Research Institute (IARI), New Delhi, India.

### Surface sterilization, seed imbibition, and half-seed preparation

The surface sterilization of seeds was performed by following the method of Di et al. (1996). Disinfected seeds were soaked in Erlenmeyer flask containing sterile distilled water, and incubated in an orbital shaker at 120 rpm under total darkness for 1 day at 25 ± 2 °C. A longitudinal cut was made in imbibed seeds through the hilum to separate cotyledons, and seed coat was removed. One-half of seed (cotyledon with embryonic axis attached) was used in the experiment. The plumule and radicle tip were dissected to obtain half-seed explant.

### Optimization of shoot induction

Half-seed explants were cultured (flat side up and radical portion embedded in the medium) on shoot induction medium (SIM) containing various concentrations of cytokinins, BA (1.11–8.88 μM), kinetin (KT 2.33–18.60 µM), and thidiazuron (TDZ 0.46–1.82 µM) (Sigma, St. Louis, USA), individually. After 15 days of initial culture, explants were sub-cultured twice into fresh SIM at 15 days interval for shoot proliferation. Separate experiments were carried out by culturing half-seed explants on SIM containing optimal concentration of BA (4.44 μM) in combination with different concentrations of PAs, such as spermidine (34.42–172.11 μM), spermine (24.71–123.55 μM), and putrescine (31.04–155.20 μM) (Sisco Research Laboratories, Mumbai, India).

### Optimization of shoot elongation

Half-seed explants with shoots after 45 days of culture on optimal SIM (4.44-µM BA and 103.27-µM spermidine) were transferred to shoot elongation medium (SEM) supplemented with various concentrations of GA_3_ (0.72–5.78 µM), zeatin riboside (ZTR 2.29–11.41 µM), and indole-3-acetic acid (IAA 0.58–2.86 µM) (Sigma, St. Louis, USA) individually. After 15 days of culture, one subculture was done with fresh SEM and incubated for another 15 days. Separate experiments were carried out by culturing explants with multiple shoots on SEM containing optimal concentration of GA_3_ (1.45 μM) in combination with different concentrations of PAs as mentioned above.

### Optimization of rooting and plant acclimatization

Elongated shoots, which attained a height of 4 cm and above, were excised, and cultured on root induction medium (RM) containing various concentrations of IBA (2.47–12.31 µM) (Sigma, St. Louis, USA), and PAs (as mentioned above) individually for 30 days. Rooted plantlets were removed from the culture tubes and washed with running tap water to remove gelling agent from the root surface. The plantlets were then transferred to plastic cups containing sterile sand, soil, and vermiculate (1:1:1 v/v/v) and grown in growth chamber at 25 ± 2 °C with 85 % relative humidity (RH) for 2–3 weeks. Upon growth, the plantlets were transferred to earthen pots containing sterile sand, soil, and vermiculate (1:1:1 v/v/v) and grown in the greenhouse under controlled conditions.

### Basal media, controls, and culture condition

The basal medium used in optimization was MSB_5_ [MS salts, MSIII iron, and B_5_ vitamins (Murashige and Skoogs 1962; Gamborg et al. 1968)] supplemented with 3-mM 2-(*N*-morpholino)ethanesulfonic acid (MES), 87.65-mM sucrose, and 0.2 % phytagel (pH 5.8). Controls for shoot induction, elongation, and rooting were maintained on hormone-free MSB_5_ medium. All the cultures unless specified were incubated at 25 ± 2 °C under a 16-h photoperiod (50 μmol m^−2^ s^−1^) provided by cool white fluorescent lamps (Philips, Delhi, India).

### *Agrobacterium* strain and binary vector

**Fig. 1:**
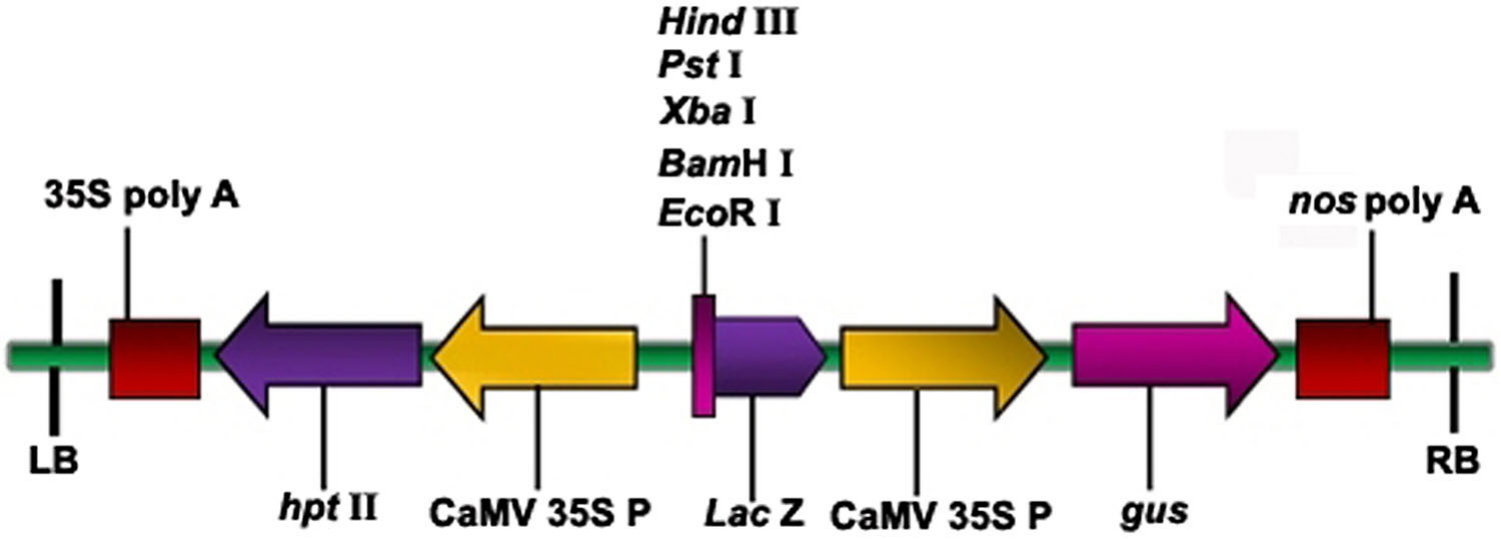
Schematic representation of the binary vector pCAMBIA1301 used in half-seed transformation of soybean cv. DS 97–12. The T-DNA region of pCAMBIA1301 showing the assembly of *hpt* II gene expression cassette (CaMV 35S P: *hpt* II: 35S poly A) and *gus* gene expression cassette (CaMV 35S P: *gus*: *nos* poly A). *CaMV* *35S P* cauliflower mosaic virus 35S promoter*, hpt* II hygromycin phosphotransferase II, *35S poly A* cauliflower mosaic virus 35S poly A terminator, *gus* *β*-glucuronidase gene, *nos* *poly A* nopaline synthase poly A terminator

*Agrobacterium tumefaciens* strain EHA105 harboring the binary vector pCAMBIA1301 (Fig. 1) was used in the present study. The T-DNA region of the binary vector contains cauliflower mosaic virus 35S (CaMV 35S) promoter-driven *hygromycin phosphotransferase* II (*hpt* II) gene, and *β*-*glucuronidase* (*gus*) gene as plant selection and reporter markers, respectively (Fig. 1). The backbone of the vector carries *neomycin phosphotransferase* II (*npt* II) gene for bacterial selection.

### Assessment on influence of PAs on transformed plant production

The plant transformation was carried out by sonication and vacuum infiltration-assisted *Agrobacterium*-mediated genetic transformation as described by Arun et al. (2015) with slight modifications. Half-seed explants of cv. DS 97–12 after agroinfection were placed (adaxial side down) on co-cultivation medium (CCM) containing 4.44 µM BA. After 5 days of dark incubation, explants were washed, blot dried, and inoculated into SIM containing 4.44-µM BA, 200-mg l^−1^ cefotaxime, and 50-mg l^−1^ vancomycin for first 15 days. After 15 days, the explants were transferred to SIM amended with aforesaid antibiotics along with 10-mg l^−1^ hygromycin B and sub-cultured twice at 15 days interval. Half-seeds with developed shoots were then transferred to SEM containing 1.45-µM GA_3_, 100-mg l^−1^ cefotaxime, 25-mg l^−1^ vancomycin, and 10-mg l^−1^ hygromycin B. The explants with shoots were sub-cultured in SEM at 15 days interval. After 30 days, the elongated shoots were separated, inoculated into RM containing 4.93-µM IBA and 4-mg l^−1^ hygromycin B, and cultured for 30 days. Separate experiments were carried out by co-cultivating agroinfected explants in CCM containing 4.44-µM BA and 103.27-µM spermidine. The co-cultivated explants were then washed and regenerated in SIM containing 4.44-µM BA and 103.27-µM spermidine, SEM containing 1.45-µM GA_3_ and 49.42-µM spermine, and RM containing 62.08-µM Putrescine. Sub-cultures and selection were carried out as mentioned above. The rooted plantlets were acclimatized in growth chamber and transferred to greenhouse upon growth.

### GUS histochemical assay

GUS histochemical assay was performed according to the method described by Jefferson et al. (1987). Leaves, stem, and pods from all the putatively transformed plants along with respective controls from wild-type (WT) plants were incubated overnight at 37 °C in 2-mM X-Gluc (5-bromo-4-chloro-3-indolyl β-d-glucuronide) in a phosphate buffer (pH 7.0) containing 10-mM EDTA, 0.5-mM potassium ferricyanide, 0.5-mM potassium ferrocyanide, and 0.1 % (v/v) Triton X-100. The chlorophyll was removed from the plant tissues using 95 % methanol after X-Gluc staining.

### Molecular analysis of transgenic plants

Five randomly selected GUS-positive plants were used for molecular analysis. The PCR analysis was carried out on genomic DNA using a set of forward primer *hpt* II FP: 5-GATGTTGGCGACCTCGTATT-3 and reverse primer *hpt* II RP: 5-GTGTCACGTTGCAAGACCTG-3 to amplify a 407-bp fragment of the *hpt* II gene. Plasmid pCAMBIA1301 and WT plant DNA were used as positive and negative controls, respectively. The PCR was initiated in a PTC-100^TM^ thermal cycler (MJ research Inc, Waltham, USA) programmed with a hot start at 94 °C for 4 min, followed by 30 cycles of 94 °C for 1 min, 55 °C for 1 min, and 72 °C for 1 min, followed by a final extension at 72 °C for 7 min. The amplified fragments were analyzed by electrophoresis at 50 V on a 1 % (w/v) agarose (SRL, Mumbai, India) gel.

Southern blot hybridization was performed to detect the *hpt* II gene integration and copy number. Ten micrograms of the genomic DNA from PCR-positive, WT soybean plants, and 5 μg of pCAMBIA1301 plasmid were digested with *Eco*RI and fragments were size-fractionated on a 1.0 % (w/v) agarose gel, and subsequently transferred to a Hybond N+ membrane (GE healthcare, Buckinghamshire, UK). The membrane was hybridized with a probe prepared by labeling the PCR purified product (407 bp) of *hpt* II gene using AlkPhos Direct Labeling kit (GE Healthcare Limited, Buckinghamshire, England). The probe was detected in the membrane according to the manufacturer’s instructions.

### Statistical analysis

For multiple shoot induction, 50 explants were cultured per treatment. For shoot elongation, 50 explants with multiple shoots were cultured per treatment. For rooting, 50 elongated shoots which attained 4 cm height were cultured per treatment. For *Agrobacterium*-mediated transformation, 100 explants were infected per treatment. Each treatment was repeated thrice, and data were statistically analyzed using analysis of variance (ANOVA). Data are presented as mean (±) standard error. The mean separations were carried out using Duncan’s multiple range test, and significance was determined at 5 % level (SPSS 11.5).

## Results and discussion

**Fig. 2:**
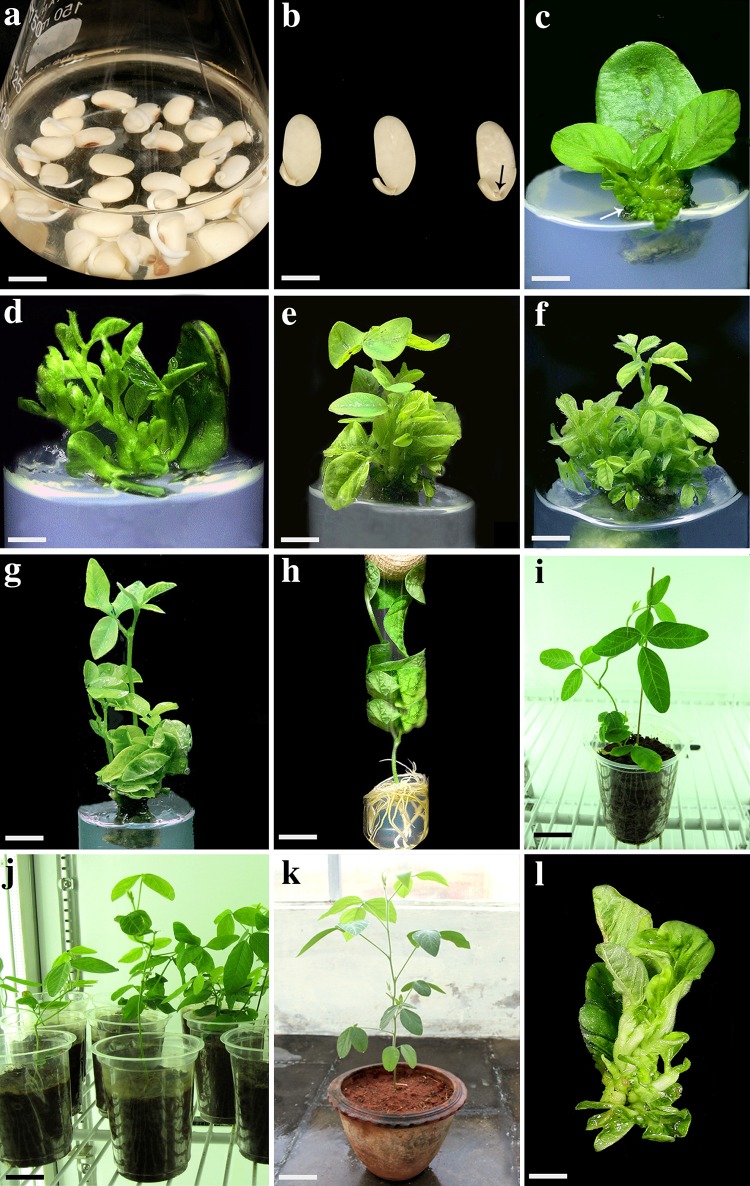
Effect of exogenous polyamines on direct regeneration and in vitro rooting using half-seed explants of soybean cv. DS 97–12. **a** One-day-old imbibed seeds; **b** half-seed explants prepared from imbibed seeds (*black arrow* indicate the meristematic region); **c–f** axillary shoot induction (*white arrow* indicates the axillary shoot), proliferation, and shoot development on SIM containing BA (4.44 µM) and spermidine (103.27 µM); **g** elongated shoots on SEM containing GA_3_ (1.45 µM) and spermine (49.42 µM) after 30 days of culture; **h** rooted shoot on RM containing putrescine (62.08 µM) after 30 days of culture; **i, j** hardened plants in plastic cups; **k** plant grown in greenhouse; **l** explant with fasciated shoots on SIM containing TDZ (0.46 µM). *Bars* 5 mm (**a**, **b**); 10 mm (**c**–**l**)

The positive influence of exogenous PAs on plant regeneration has been reported in several crops. In addition, PAs are also well studied to assess their participation on increasing plant tolerance against different abiotic stresses. However, until now, information regarding PAs involvement on improving plant transformation is less focused. Hence, in the present study, an attempt was made to investigate the influence of PAs on direct regeneration and *Agrobacterium*-mediated plant transformation of soybean. Half-seeds (Fig. 2b) derived from 1-day-old imbibed seeds (Fig. 2a) were used as explants for this study.

### Optimization of shoot induction

In the present study, choice of cytokinins showed significant influence on shoot regeneration from half-seeds. The results confirmed that BA was more effective in inducing multiple shoots than KN and TDZ (Supplementary Table 1). At an optimal concentration (4.44 µM), BA displayed a superior effect on shoot production (74.6 %) and produced an average of 15.6 shoots/explant after 45 days of culture. These results suggest that, BA could have exerted an enhanced cytokinins activity during meristematic cell differentiation of half-seeds compared with KN and TDZ. The superiority of BA on shoot induction over other PGRs, such as IBA and KT (Ma and Wu 2008), and ZTR and KT (Zia et al. 2010a), was reported in soybean. In the case of KN, an optimal concentration of 4.65 µM showed maximum response (66.0 %) on shoot production (9.6 shoots/explant). Even though TDZ (0.46–1.82 µM) showed multiple shoot development, the shoots were mostly abnormal in appearance. Abnormalities, such as fasciations and shoots in rosettes, are mainly due to the phenyl group of TDZ, and are reported as heritable traits commonly associated with a long-term use of TDZ (Preece et al. 1987; Huetteman and Preece 1993). Our observations confirmed that the usage of TDZ even at very low concentration (0.46 µM) resulted in the production of shoots in rosettes from half-seed explants (Fig. 2l). In contrast to the present results, Kaneda et al. (1997) reported multiple shoots regeneration with normal shoot morphology from cotyledonary nodes and hypocotyl segments of soybean on media supplemented with high concentration of TDZ (9 µM). Also, Janani and Ranjitha Kumari (2013) achieved maximum number of shoots with no abnormalities from cotyledonary nodes of soybean using 15-μM TDZ. This discrepancy in action of TDZ might be due to the choice of explant preferred in each study and/or genotypic effect that exists between the soybean cultivars.

In the present study, exogenous application of PAs in combination with SIM containing optimal concentration of BA (4.44 µM) exhibited a synergistic effect and resulted in high-frequency shoot induction from half-seed explants (Table 1). Among the different combinations of BA and spermidine tested, spermidine at 103.27 μM was found optimal and showed the highest percentage of response (98.3 %) with increased production of multiple shoots (36.6 shoots/explant) after 45 days of culture (Table 1; Fig. 2c–f). The possible interaction of meristematic cells between spermidine (nitrogen donor), BA (a potent cytokinin), and reserve food materials available within soybean cotyledons (Franklin et al. 2004) could be the reason for this increased morphogenetic response in half-seeds. Furthermore, the maximum percentage of response and number of shoots achieved in this study using optimal BA (4.44 µM) and spermidine (103.27 μM) was significantly higher compared with earlier reports on soybean half-seed regeneration system involving only PGRs (Radhakrishnan and Ranjitha Kumari 2007; Zia et al. 2010a; Lee et al. 2011; Ether et al. 2013; Janani and Ranjitha Kumari 2013). Our results related to spermidine are in agreement with the report of Khanna and Daggard (2001) in wheat. The authors supplemented medium with 100-µM spermidine to achieve enhanced plant regeneration from wheat calli. Similar results with spermidine on improving shoot regeneration have been reported in *Torenia* (Tanimoto et al. 1994). Although the addition of spermine (74.13 μM) and putrescine (93.12 µM) in SIM containing BA (4.44 µM) produced higher percentage of response as well as number of shoots/explant when compared with BA alone, the results obtained were not as significant as spermidine (Table 1). In *Cucumis sativus* (Vasudevan et al. 2008) and *Withania somnifera* (Sivanandhan et al. 2011), supplementation of spermidine to regeneration medium achieved enhanced shoot regeneration compared with spermine and putrescine as observed in the present study. The shoots regenerated in all tested BA and PAs combinations were healthy, exhibited normal morphology and, however, failed to elongate on the same shoot induction media.

**Table 1:** Effect of polyamines on multiple shoot induction from half-seed explants (derived from 1-day-old imbibed seeds) of soybean cv. DS 97–12 on SIM containing BA (4.44 µM) after 45 days of culture

Polyamines (μM)	Percentage of explant responding (%)	Mean number of shoots/explants
BA^a^		
4.44	74.6 ± 0.3l	15.6 ± 0.2l
BA + spermidine^b^		
34.42	89.0 ± 0.3e	28.0 ± 0.4e
68.84	93.0 ± 0.4c	31.6 ± 0.3c
103.27	98.3 ± 0.4a	36.6 ± 0.2a
137.69	95.3 ± 0.4b	33.3 ± 0.3b
172.11	91.3 ± 0.5d	29.6 ± 0.4d
BA + spermine^c^		
24.71	82.6 ± 0.5h	22.0 ± 0.5hi
49.42	84.3 ± 0.3g	25.6 ± 0.3f
74.13	89.3 ± 0.6e	30.6 ± 0.4cd
98.84	87.3 ± 0.3f	27.6 ± 0.4e
123.55	83.6 ± 0.4gh	24.0 ± 0.3g
BA + putrescine^d^		
31.04	81.0 ± 0.3i	22.6 ± 0.3h
62.08	83.3 ± 0.6gh	25.3 ± 0.2f
93.12	84.6 ± 0.4g	27.3 ± 0.5e
124.16	80.6 ± 0.5ij	21.3 ± 0.4ij
155.20	76.6 ± 0.3k	18.3 ± 0.4k

Values represent the mean (±) standard error of three independent experiments. Mean values followed by the different letters within a column are significantly different according to Duncan’s multiple range test (DMRT) at 5 % level

^a^Half-seed explants cultured on SIM containing optimal concentration of BA (4.44 µM)

^b^Half-seed explants cultured on SIM containing optimal concentration of BA (4.44 µM) and spermidine (34.42–172.11 μM)

^c^Half-seed explants cultured on SIM containing optimal concentration of BA (4.44 µM) and spermine (24.71–123.55 μM)

^d^Half-seed explants cultured on SIM containing optimal concentration of BA (4.44 µM) and putrescine (31.04–155.20 μM)

### Optimization of shoot elongation

Among the different developmental stages in direct regeneration, shoot elongation phase is acute for legume regeneration. Hence, improving shoot elongation efficiency and length are pivotal to attain improved regeneration in any explant/genotype. In the present investigation, shoots developed from half-seed explants on SIM containing BA (4.44 μM) and spermidine (103.27 μM) were subjected to shoot elongation. The SEM containing optimal concentration of GA_3_ (1.45 µM) resulted in 69.6 % of response for half-seeds after 30 days of culture, and at this concentration, 15.3 elongated shoots/explant were obtained with an average shoot length of 6.3 cm (Supplementary Table 2). In a similar study, supplementation of GA_3_ alone showed a positive influence on elongation of multiple shoots derived from half-seed explants of soybean (Janani and Ranjitha Kumari 2013). In soybean mature and immature cotyledon regeneration, Franklin et al. (2004) achieved efficient shoot elongation in MS medium containing only GA_3_. In chickpea, Jayanand et al. (2003) reported that application of GA_3_ played a critical role in shoot elongation by improving internodal length and leaf morphology. In contrast to the present study, multiple shoots regenerated from soybean half-seed failed to elongate on medium containing BA and GA_3_ (Ether et al. 2013). This could be due to the difference in tested genotypes. In this study, next to GA_3_, explants cultured on SEM containing ZTR showed better results for shoot elongation followed by IAA (Supplementary Table 2).

In the present investigation, the addition of PAs to SEM containing optimum concentration of GA_3_ (1.45 µM) also showed a positive correlation in terms of shoot elongation. The maximum response was shown by GA_3_ and spermine combination (Table 2). In SEM containing GA_3_ (1.45 µM) and spermine (49.42 µM), the percentage of response was increased up to 90.0 %, and 27.3 elongated shoots/explants were produced with an average shoot length of 8.1 cm after 30 days of culture (Table 2; Fig. 2g). In the present study, spermidine and putrescine combinations with GA_3_ (1.45 µM) also generated better response when compared with individual treatment of GA_3_ (Table 2). The positive influence of polyamines on shoot elongation could be attributed to their stimulatory effect on cell division as suggested by Bais and Ravishankar (2002). In a similar study, Nas (2004) reported that, inclusion of polyamines to the culture medium improved shoot elongation by 83 % and increased buds per shoot by 41 % in Hazelnut.

**Table 2:** Effect of polyamines on shoot elongation of regenerated shoots from half-seed explants (derived from 1-day-old imbibed seeds) of soybean cv. DS 97–12 on SEM containing GA_3_ (1.45 µM) after 30 days of culture

Polyamines (µM)	Percentage of explants responding (%)	Mean number of elongated shoots/explant	Mean shoot length (cm)
GA_3_^a^			
1.45	69.6 ± 0.4h	15.3 ± 0.5f	6.3 ± 0.3g
GA_3_ + spermidine^b^			
34.42	70.3 ± 0.4h	18.6 ± 0.5e	6.1 ± 0.2h
68.84	73.3 ± 0.3g	21.3 ± 0.3d	6.4 ± 0.3g
103.27	76.3 ± 0.5f	24.3 ± 0.3bc	7.2 ± 0.3d
137.69	74.0 ± 0.3g	23.0 ± 0.4c	6.8 ± 0.3f
172.11	67.6 ± 0.4i	16.0 ± 0.2f	6.0 ± 0.2h
GA_3_ + spermine^c^			
24.71	83.3 ± 0.6d	21.3 ± 0.3d	7.0 ± 0.2e
49.42	90.0 ± 0.3a	27.3 ± 0.6a	8.1 ± 0.2a
74.13	87.6 ± 0.5b	25.6 ± 0.3b	7.8 ± 0.2b
98.84	85.3 ± 0.3c	24.0 ± 0.4c	7.4 ± 0.3c
123.55	78.6 ± 0.3e	19.0 ± 0.5e	6.7 ± 0.3f
GA_3_ + putrescine^d^			
31.04	65.3 ± 0.5j	16.3 ± 0.3f	5.6 ± 0.1i
62.08	67.3 ± 0.3i	19.3 ± 0.5e	6.3 ± 0.2g
93.12	70.0 ± 0.6h	21.0 ± 0.4d	6.7 ± 0.3f
124.16	68.0 ± 0.4i	18.3 ± 0.6e	6.1 ± 0.2h
155.20	62.0 ± 0.3k	13.0 ± 0.4g	5.0 ± 0.2j

Values represent the mean (±) standard error of three independent experiments. Mean values followed by the different letters within a column are significantly different according to Duncan’s multiple range test (DMRT) at 5 % level

^a^Half-seed explants with regenerated shoots cultured on SEM containing optimal concentration of GA_3_ (1.45 µM)

^b^Half-seed explants with regenerated shoots cultured on SEM containing optimal concentration of GA_3_ (1.45 µM) and spermidine (34.42–172.11 μM)

^c^Half-seed explants with regenerated shoots cultured on SEM containing optimal concentration of GA_3_ (1.45 µM) and spermine (24.71–123.55 μM)

^d^Half-seed explants with regenerated shoots cultured on SEM containing optimal concentration of GA_3_ (1.45 µM) and putrescine (31.04–155.20 μM)

### Optimization of rooting

In the present investigation, elongated shoots derived from half-seed explants produced 6.3 roots/shoot with mean root length of 9.6 cm in RM containing optimal concentration of IBA (4.93 µM) after 30 days of culture (Supplementary Table 3). The rooting response was found to be 83.3 % (Supplementary Table 3). On the other hand, RM containing putrescine alone (62.08 µM) showed the highest rooting percentage of 96.3 % and developed 9.0 roots/shoot. The root length was found to be 14.6 cm after 30 days of culture (Table 3; Fig. 2h). The results obtained using optimal concentration of putrescine (62.08 µM) were significantly higher as compared with RM containing optimal concentration of IBA (4.93 µM). Also, all concentrations of spermidine and spermine employed for rooting resulted with callus formation at the bottom of shoot, which hindered the root formation completely. These observations indicate that, in soybean, the rooting potential could be confined only to putrescine as witnessed in our earlier report using cotyledonary nodes (Arun et al. 2014). In a similar study, Sivanandhan et al. (2011) reported that putrescine alone improved rooting efficiency in *W. somnifera,* whereas spermine and spermidine showed no response to root induction. Denaxa et al. (2014) claimed that putrescine application enhanced the rooting response of difficult-to-root ‘Kalamata’ olive cultivar, whereas spermidine and spermine failed to promote rooting. Free endogenous putrescine is considered as a marker of *in vitro* root induction, and its catabolism could be essential for root development by supplying H_2_O_2_ (Hausman et al. 1997; Neves et al. 2002). It has been previously shown that exogenously applied putrescine simultaneously increase the accumulation of endogenous putrescine to promote root growth (Tanguy and Carre 1993; Hausman et al. 1995). It has also been reported that putrescine acts as a second messenger, correlating with the peak of root mitotic activity (Tiburcio et al. 1989). In the present investigation, rooted plantlets are hardened and grown in growth chamber for 2–3 weeks (Fig. 2i, j). Upon growth, the plantlets were transferred to greenhouse and grown under controlled conditions (Fig. 2k).

**Table 3:** Effect of polyamines on rooting of elongated shoots from half-seed explants of soybean cv. DS 97–12 on RM after 30 days of culture

Polyamines (µM)	Rooting response (%)	Mean number of roots/shoot	Mean root length (cm)
IBA^a^			
4.93	83.3 ± 0.6c	6.3 ± 0.4c	9.6 ± 0.2c
Spermidine^b^			
34.42	0.0g	0.0f	0.0f
68.84	0.0g	0.0f	0.0f
103.27	0.0g	0.0f	0.0f
137.69	0.0g	0.0f	0.0f
172.11	0.0g	0.0f	0.0f
Spermine^c^			
24.71	0.0g	0.0f	0.0f
49.42	0.0g	0.0f	0.0f
74.13	0.0g	0.0f	0.0f
98.84	0.0g	0.0f	0.0f
123.55	0.0g	0.0f	0.0f
Putrescine^d^			
31.04	93.6 ± 0.5b	8.0 ± 0.4b	12.0 ± 0.3b
62.08	96.3 ± 0.3a	9.0 ± 0.3a	14.6 ± 0.2a
93.12	80.6 ± 0.5d	6.6 ± 0.5c	9.3 ± 0.1c
124.16	77.6 ± 0.6e	5.0 ± 0.6d	7.6 ± 0.2d
155.20	73.6 ± 0.5f	3.6 ± 0.4e	5.6 ± 0.1e

Values represent the mean (±) standard error of three independent experiments. Mean values followed by the different letters within a column are significantly different according to Duncan’s multiple range test (DMRT) at 5 % level

Elongated shoots above 4 cm were used for all the treatments

^a^Elongated shoots cultured on RM containing optimal concentration of IBA (4.93 µM)

^b^Elongated shoots cultured on RM containing spermidine (34.42–172.11 μM)

^c^Elongated shoots cultured on RM containing spermine (24.71–123.55 μM)

^d^Elongated shoots cultured on RM containing putrescine (31.04–155.20 μM)

The regeneration, elongation, and rooting efficiency obtained with the optimized protocol in the present study using half-seed explants (cv. DS 97–12) were found to be most promising as observed in our earlier report on soybean cotyledonary nodes (cv. PK 416) using PAs (Arun et al. 2014). Overall results of both studies thus validate the positive influence of PAs on plant regeneration in different explants/genotypes of soybean.

### Assessment on influence of PAs on transformed plant production

In the present investigation, transformed half-seed explants co-cultivated and regenerated in medium (CCM and SIM) containing 4.44-µM BA and 103.27-µM spermidine showed an improved response toward shoot induction (74.3 explants responded) and produced a total number of 541.3 shoots. A total number of 343.6 elongated shoots were obtained with SEM containing 1.45-µM GA_3_ and 49.42-µM spermine. In RM containing putrescine (62.08 µM), a total number of 161.6 elongated shoots developed roots. Finally, 99.6 putative transformants survived after hardening (Table 4). On the other hand, 48.6 explants responded and regenerated a total number of 313.0 shoots in CCM and SIM containing only BA (4.44 µM). Using SEM containing only GA_3_ (1.45 µM), a total number of 149.3 elongated shoots were achieved. Also, a total number of 77.6 rooted shoots were obtained on treatment with IBA (4.93 µM) alone. Furthermore, a total number of 39.3 putative transformants survived after hardening (Table 4). The GUS expression analysis was carried out in all putative transformants that are grown to maturity under greenhouse condition. Based on GUS analysis, optimized protocol (comprising PAs and PGRs) showed improved transformation efficiency (29.3 %) compared with the regeneration system involving only PGRs (14.6 %) (Table 4).

**Table 4:** *Agrobacterium*-mediated transformation of half-seed explants of cv. DS 97–12 transformed with EHA105 harboring pCAMBIA1301 plasmid

Experimental set up	Number of explants infected	Mean number of explants responded	Mean number of shoots produced	Mean number of elongated shoots	Mean number of rooted shoots	Mean number of plants survived	Mean number of GUS^+^ plants	Transformation efficiency (%)^c^
PAs (−)^a^	100	48.6 ± 0.8b	313.0 ± 0.6b	149.3 ± 0.3b	77.6 ± 0.4b	39.3 ± 0.7b	14.6 ± 0.3b	14.6 ± 0.3b
PAs (+)^b^	100	74.3 ± 0.5a	541.3 ± 0.4a	343.6 ± 0.7a	161.6 ± 0.6a	99.6 ± 0.6a	29.3 ± 0.5a	29.3 ± 0.5a

Mean values of three independent experiments (±) with standard errors. Values with the different letters within columns are significantly different according to Duncan’s multiple range test (DMRT) at a 5 % level

^a^The half-seed explants were sonicated for 20 s, vacuum infiltered for 2 min at 250 mm of Hg, co-cultivated in CCM (4.44-µM BA) and regenerated in SIM containing 4.44-µM BA, SEM containing 1.45-µM GA_3_, and RM containing 4.93-µM IBA

^b^The half-seed explants were sonicated for 20 s, vacuum infiltered for 2 min at 250 mm of Hg, co-cultivated in CCM (4.44-µM BA and 103.27-µM spermidine), and regenerated in SIM containing 4.44-µM BA, 103.27-µM spermidine, SEM containing 1.45-µM GA_3_, 49.42-µM spermine, and RM containing 62.08-µM putrescine

^c^Transformation efficiency = number of GUS^+^ plants/total no of infected explants ×100

Increasing adventitious regeneration and competence in transformation are acute for the application of genetic engineering techniques (Petri et al. 2005). Results of our study confirmed that, involvement of PAs significantly benefited transformed plant regeneration at all the developmental stages. In the present study, possible explanation on improved production of transformed shoots by BA and spermidine could be due to its synergistic effect involved in inducing sufficient amount of dividing target cells that are competent for *Agrobacterium* to easily transfer its T-DNA. This synergistic combination could also support meristematic region to retain its regeneration potential by alleviating stress induced by infection and selection procedures, which improved shoot production. In addition, spermidine might also increase *vir* gene induction to enhance T-DNA transfer in half-seed competent cells as observed in tobacco (Kumar and Rajam 2005). Moreover, a positive correlation between GA_3_ and spermine significantly improved shoot elongation. Also, putrescine developed roots with increased length that aided in high survival of putative transformants. Overall, these optimal conditions were highly supportive in regeneration of healthy transformed plants and improved transformation efficiency (29.3 %). On the other hand, we were not able achieve these advantages on transgenic plant production by regenerating explants in a system involving only PGRs. Thus, the results of the present study validate the positive role played by PAs in improving transformation efficiency. In a similar study, inclusion of 100 µM spermidine in regeneration media of infected wheat calli showed improved recovery of transformants (7–24.2 %) and increased transformation frequency (1.2–3.9 %) compared with control (Khanna and Daggard 2003).

In previous reports, exogenous application of PAs enhanced plant tolerance to several abiotic stresses, such as salinity (Kamiab et al. 2014), drought (Yin et al. 2014), heat (Mostofa et al. 2014), and heavy metals (Sun et al. 2014). Therefore, transformed plantlets regenerated in the present study using PAs are expected to display increased resistance toward abiotic stress as observed in previous reports. This hypothesis was supported with recent reports in soybean (Radhakrishnan and Lee 2013a, b). The authors reported that, exogenous application of spermine counteracted osmotic stress in soybean and improved pod/seed quality.

### Transgene expression and molecular characterization of transformed soybean plants

**Fig. 3:**
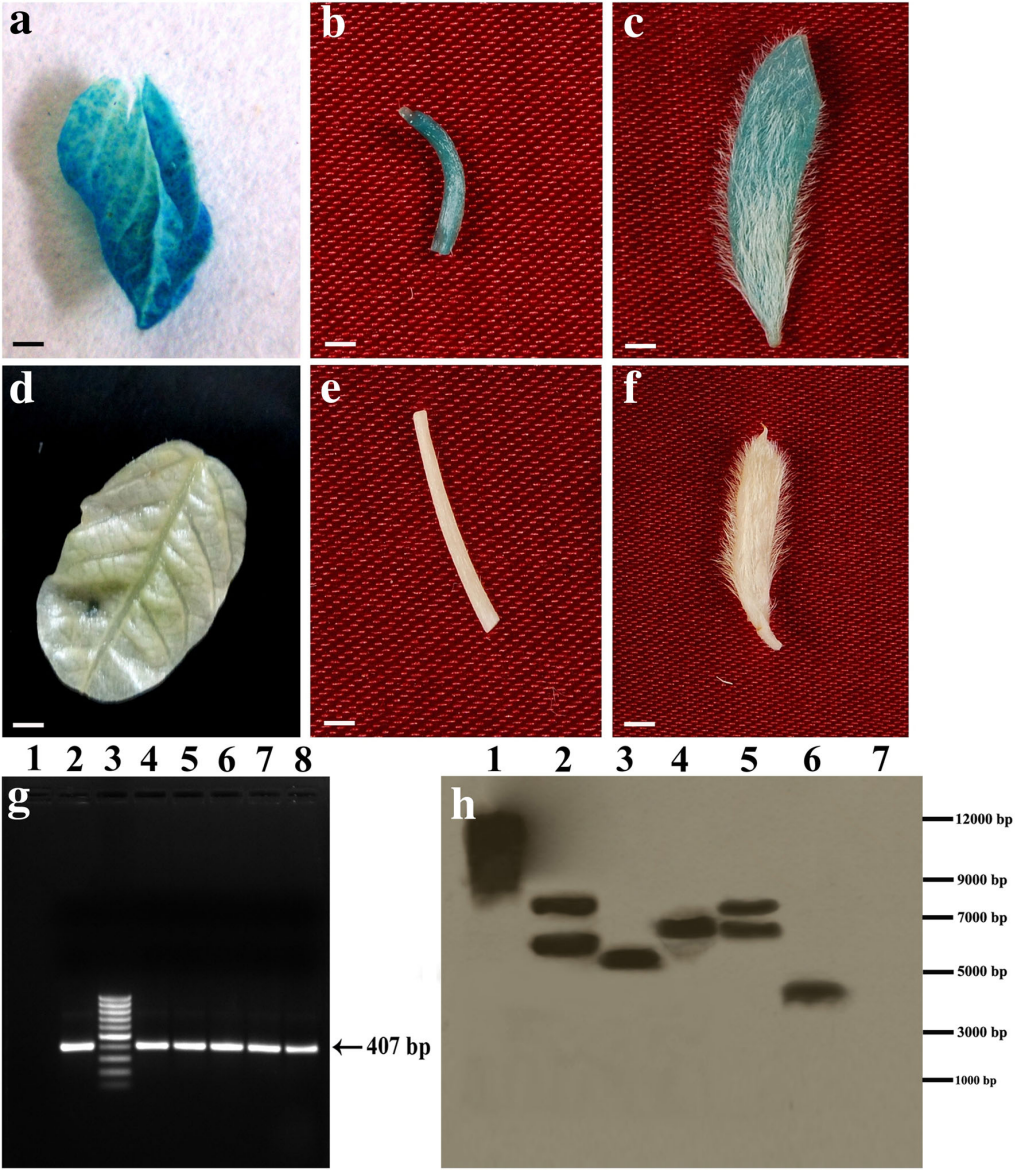
Transgene expression and molecular confirmation of putative transformants regenerated from half-seed explants of cv. DS 97–12 infected with *A. tumefaciens* strain EHA105 harboring pCAMBIA1301. **a** Expression of *gus* gene in transformed mature leaf; **b** expression of *gus* gene in transformed stem segment; **c** transformed soybean pod showing *gus* gene expression; **d** leaf from WT plant; **e** stem segment from WT plant; **f** pod from WT plant; **g** PCR analysis of transformants for detection of *hpt* II gene. *Lane 1* DNA sample from WT plant as negative control, *lane 2* pCAMBIA1301 as a positive control, *lane 3* 100 bp plus DNA ladder, *lanes 4*–*8* DNA samples of transformants (arrow indicates the amplification of *hpt* II gene at 407 bp); **h** Southern blot hybridization of genomic DNA isolated from PCR-positive transformed plants. *Lane 1* plasmid of pCAMBIA1301 as positive control, *lanes 2*–*6* DNA sample of transformants, *lane 7* DNA from WT plant. *Bars* 5 mm (**a**–**f**)

The results of GUS assay showed an intense blue color in transformed leaves (Fig. 3a), stem (Fig. 3b), and pods (Fig. 3c). On the other hand, there was no blue coloration in counterparts from WT plants, such as leaves (Fig. 3d), stem (Fig. 3e), and pods (Fig. 3f) which served as controls. Five randomly selected GUS-positive soybean plants were subjected to analysis by PCR. The presence of expected 407-bp amplified product in all transformed GUS-positive plants genomic DNA (Fig. 3g, lanes 4–8), and pCAMBIA1301 plasmid DNA (Fig. 3g, lane 2) indicated the presence of *hpt* II gene in the soybean genome. Conversely, genomic DNA from WT soybean plant did not show any amplified product with *hpt* II specific primers (Fig. 3g, lane 1). Transgene integration and copy numbers were detected by Southern hybridization. The transformed plants exhibited simple hybridization patterns that ranged from single to three copy numbers (Fig. 3h, lanes 2–6). Also, DNA from WT plant showed no hybridization (Fig. 3h, lane 7), while pCAMBIA1301 plasmid generated hybridization signal (Fig. 3h, lane 1). The hybridization patterns were non-identical due to different transformation events.

## Conclusion

In the present investigation, involvement of PAs significantly improved both regeneration and gene transformation in half-seeds of soybean. The PAs in combination with PGRs (shoot induction and elongation phase) or alone (rooting phase) enhanced shoot induction, elongation, and rooting efficiency. Moreover, the transformation efficiency was significantly improved (29.3 %) when transformed half-seed explants were regenerated in a media setup containing optimal PAs and PGRs, compared with its counterparts (14.6 %) with only PGRs. Also, the supplementation of PAs delivered most suitable conditions for transformed plant production and subsequent regeneration. Hence, this PAs supplemented optimized transformation system can be adopted to develop elite verities in soybean that are recalcitrant to regeneration a transformation. In addition, the transformed plants regenerated in presence of PAs could also show enhanced tolerance to abiotic stress.

## Electronic supplementary material

Below is the link to the electronic supplementary material.

Supplementary material 1 (DOC 51 kb)

## References

[CR1] Arun M, Subramanyam K, Theboral J, Ganapathi A, Manickavasagam M (2014). Optimized shoot regeneration for Indian soybean: the influence of exogenous polyamines. Plant Cell Tissue Organ Cult.

[CR2] Arun M, Subramanyam K, Mariashibu TS, Theboral J, Shivanandhan G, Manickavasagam M, Ganapathi A (2015). Application of sonication in combination with vacuum infiltration enhances the *Agrobacterium*-mediated genetic transformation in Indian soybean cultivars. Appl Biochem Biotechnol.

[CR3] Bais HP, Ravishankar GA (2002). Role of polyamines in the ontogeny of plants and their biotechnological applications. Plant Cell Tissue Organ Cult.

[CR4] Curtis IS, Nam HG, Sakamoto K (2004). Optimized shoot regeneration system for the commercial Korean radish ‘Jin Ju Dae Pyong’. Plant Cell Tissue Organ Cult.

[CR5] Denaxa NK, Roussos PA, Vemmos SN (2014). The possible role of polyamines to the recalcitrance of ‘‘Kalamata’’ olive leafy cuttings to root. J Plant Growth Regul.

[CR6] Di R, Purcell V, Collins GB, Ghabrial SA (1996). Production of transgenic soybean lines expressing the bean pod mottle virus coat protein precursor gene. Plant Cell Rep.

[CR7] Ether YB, Jadhav PV, Moharil MP, Dudhare MS, Kale P, Nandanwar RS, Mane SS, Dani R (2013). Epigenesis through in vitro regeneration in soybean amenable to genetic transformation. VEGETOS.

[CR8] Franklin G, Carpenter L, Davis E, Reddy C, Al-Abed D, Abou Alaiwi W, Parani M, Smith B, Sairam R (2004). Factors influencing regeneration of soybean from mature and immature cotyledons. Plant Growth Regul.

[CR9] Gamborg OL, Miller RA, Ojiama K (1968). Nutrient requirements of suspension cultures of soybean root cells. Exp Cell Res.

[CR10] Hausman JF, Kevers C, Gaspar T (1995). Auxin-polyamine interaction in the control of the rooting inductive phase of poplar shoots in vitro. Plant Sci.

[CR100] Hausman JF, Kevers C, Evers D, Gaspar T (1997) Conversion of putrescine into γ-aminobutyric acid, an essential pathway for root formation by poplar shoots in vitro. In: Altman A, Waisel Y (eds) Biology of root formation and development. Plenum Press, New York, pp 133–140

[CR11] Huetteman CA, Preece JE (1993). Thidiazuron: a potent cytokinin for woody plant tissue culture. Plant Cell Tissue Organ Cult.

[CR12] Janani C, Ranjitha Kumari BD (2013). In vitro plant regeneration from cotyledonary node and half seed explants of *Glycine max* L. (JS335). Ann Biol Res.

[CR13] Jayanand B, Sudarsanam G, Sharma KK (2003). An efficient protocol for the regeneration of whole plants of chickpea (*Cicer arietinum* L.) by using axillary meristem explants derived from In vitro-germinated seedlings. In Vitro Cell Dev Biol Plant.

[CR14] Jefferson RA, Kavanagh TA, Bevan MW (1987). GUS fusions: β-glucuronidase as a sensitive and versatile gene fusion marker in higher plants. EMBO J.

[CR15] Kakkar RK, Nagar PK, Ahuja PS, Rai VK (2000). Polyamines and plant morphogenesis. Biol Plant.

[CR16] Kamiab F, Talaie A, Khezri M, Javanshah A (2014). Exogenous application of free polyamines enhance salt tolerance of pistachio (*Pistacia vera* L.) seedlings. Plant Growth Regul.

[CR17] Kaneda Y, Tabei Y, Nishimura S, Harada K, Akihama T, Kitamura K (1997). Combination of thidiazuron and basal media with low salt concentrations increases the frequency of shoot organogenesis in soybeans [*Glycine max* (L.) Merrill]. Plant Cell Rep.

[CR18] Kevers C, Le Gal N, Monteiro M, Dommes J, Gaspar J (2000). Somatic embryogenesis of *Panax ginseng* in liquid cultures: a role for polyamines and their metabolic pathways. Plant Growth Regul.

[CR19] Khanna HK, Daggard GE (2001). Enhanced shoot regeneration in nine Australian wheat cultivars by spermidine and water stress treatments. Aust J Plant Physiol.

[CR20] Khanna HK, Daggard GE (2003). *Agrobacterium tumefaciens*-mediated transformation of wheat using a superbinary vector and a polyamine-supplemented regeneration medium. Plant Cell Rep.

[CR21] Kumar SV, Rajam MV (2005). Polyamines enhance *Agrobacterium tumefaciens vir* gene induction and T-DNA transfer. Plant Sci.

[CR22] Kumar V, Sharma A, Narasimha Prasad BC, Gururaj HB, Giridhar P, Ravishankar GA (2007). Direct shoot bud induction and plant regeneration in *Capsicum frutescens* Mill: influence of polyamines and polarity. Acta Physiol Plant.

[CR23] Lee K, Yi BY, Kim KH, Kim JB, Suh SC, Woo HJ, Shin KS, Kweon SJ (2011). Development of efficient transformation protocol for soybean (*Glycine max* L.) and characterization of transgene expression after *Agrobacterium*-mediated gene transfer. J Korean Soc Appl Biol Chem.

[CR24] Ma XH, Wu TL (2008). Rapid and efficient regeneration in soybean [*Glycine max* (L.) Merrill] from whole cotyledonary node explants. Acta Physiol Plant.

[CR25] Mostofa MG, Yoshida N, Fujita M (2014). Spermidine pretreatment enhances heat tolerance in rice seedlings through modulating antioxidative and glyoxalase systems. Plant Growth Regul.

[CR26] Murashige T, Skoog F (1962). A revised medium for rapid growth and bio assays with tobacco tissue cultures. Physiol Plant.

[CR27] Nas MN (2004). Inclusion of polyamines in the medium improves shoot elongation in hazelnut (*Corylus avellana* L.) micropropagation. Turk J Agric For.

[CR28] Neves C, Santos H, Boas LV, Amâncio S (2002). Involvement of free and conjugated polyamines and free amino acids in the adventitious rooting of micropropagated cork oak and grapevine shoots. Plant Physiol Biochem.

[CR29] Paz MM, Martinez JC, Kalvig AB, Fonger TM, Wang K (2006). Improved cotyledonary node method using an alternative explant derived from mature seed for efficient *Agrobacterium*-mediated soybean transformation. Plant Cell Rep.

[CR30] Petri C, Alburquerque N, Perez-Tornero O, Burgos L (2005). Auxin pulses and a synergistic interaction between polyamines and ethylene inhibitors improve adventitious regeneration from apricot leaves and *Agrobacterium*-mediated transformation of leaf tissues. Plant Cell Tissue Organ Cult.

[CR31] Preece J, Huetteman C, Puello C, Neuman M (1987). The influence of thidiazuron on in vitro culture of woody plants. Hortic Sci.

[CR32] Pua CE, Sim EG, Chi LG, Kong FL (1996). Synergistic effect of ethylene inhibitors and putrescine on shoot regeneration from hypocotyls explants of Chinese radish (*Raphanus sativus* L var longipinnatus Bailey) *in vitro*. Plant Cell Rep.

[CR33] Radhakrishnan R, Lee IJ (2013). Ameliorative effects of spermine against osmotic stress through antioxidants and abscisic acid changes in soybean pods and seeds. Acta Physiol Plant.

[CR34] Radhakrishnan R, Lee IJ (2013). Spermine promotes acclimation to osmotic stress by modifying antioxidant, abscisic acid, and jasmonic acid signals in soybean. J Plant Growth Regul.

[CR35] Radhakrishnan R, Ranjitha kumari BD (2007). Callus induction and plant regeneration of Indian soybean (*Glycine max* (L.) Merr. cv. CO3) via half seed explant culture. J Agric Technol.

[CR36] Sivanandhan G, Mariashibu TS, Arun M, Rajesh M, Kasthurirengan S, Selvaraj N, Ganapathi A (2011). The effect of polyamines on the efficiency of multiplication and rooting of *Withania somnifera* (L.) Dunal and content of some withanolides in obtained plants. Acta Physiol Plant.

[CR37] Sun Z, Liu Y, Huang Y, Zeng G, Wang Y, Hu X, Zhou L (2014). Effects of indole-3-acetic, kinetin and spermidine assisted with EDDS on metal accumulation and tolerance mechanisms in ramie (*Boehmeria nivea* (L.) Gaud.). Ecol Eng.

[CR38] Tanguy JM, Carre M (1993). Polyamines in grapevine microcuttings cultivated in vitro. Effects of amines and inhibitors of polyamine biosynthesis on polyamine levels and microcutting growth and development. Plant Growth Regul.

[CR39] Tanimoto S, Matsubara Y, Ishioka N (1994). Significance of spermidine in the initiation of adventitious buds in stem segments of Torenia. Plant Cell Physiol.

[CR40] Tiburcio AF, Gendy CA, Van TT (1989). Morphogenesis in tobacco subepidermal cells: putrescine as marker of root differentiation. Plant Cell Tissue organ Cult.

[CR41] Vasudevan A, Selvaraj N, Ganapathi A, Kasthurirengan S, Ramesh Anbazhagan V, Manickavasagam M, Choi C (2008). Leucine and spermidine enhance shoot differentiation in cucumber (*Cucumis sativus* L.). In Vitro Cell Dev Biol Plant.

[CR42] Yin ZP, Li S, Ren J, Song XS (2014). Role of spermidine and spermine in alleviation of drought-induced oxidative stress and photosynthetic inhibition in Chinese dwarf cherry (*Cerasus humilis*) seedlings. Plant Growth Regul.

[CR43] Zia M, Rizvi ZF, Rehman R, Chaudhary MF (2010). Micropropagation of two Pakistani soybean [*Glycine max* (L.) Merrill] cultivars from cotyledon nodes. Span J Agric Res.

[CR44] Zia M, Rizvi ZF, Rehman RU, Chaudhary MF (2010). *Agrobacterium* mediated transformation of soybean (*Glycine Max* L.): some conditions standardization. Pak J Bot.

