# Supplementary material for: Involvement of exogenous polyamines enhances regeneration and Agrobacterium-mediated genetic transformation in half-seeds of soybean
Source: 3 Biotech. 2016 Jun 28;6(2):148. doi: 10.1007/s13205-016-0448-0 (PMC4925569; doi:10.1007/s13205-016-0448-0)
Supplement: Supplementary file 1 — Supplementary material 1 (DOC 51 kb) [file 13205_2016_448_MOESM1_ESM.doc]

**Supplementary table 1** Effect of cytokinins on multiple shoot induction from half-seed explants (derived from 1-day-old imbibed seeds) of soybean cv. DS 97–12 on SIM after 45 days of culture

| **Plant growth**  **regulators (μM)** | **Percentage of explant responding (%)** | **Mean number of shoots/explants** |
| --- | --- | --- |
| Control^*^  BA  1.11  2.22  4.44  6.66  8.88  KT  2.33  4.65  9.30  13.95  18.60  TDZ  0.46  0.91  1.37  1.82 | 11.6±0.2l  66.3±0.4d  71.0±0.5b  74.6±0.3a  68.6±0.6c  63.3±0.4e  61.0±0.2f  66.0±0.4d  55.3±0.3g  47.0±0.4h  42.6±0.4i  55.6±0.2g  47.6±0.4h  35.6±0.3j  21.3±0.5k | 1.3±0.1k  10.0±0.4d  13.6±0.3b  15.6±0.2a  12.0±0.4c  8.6±0.3e  7.3±0.4f  9.6±0.3d  6.3±0.5g  5.0±0.3h  3.3±0.4ij  7.3±0.2f  6.0±0.4g  4.0±0.3i  3.0±0.5j |

^*^ Half-seed explants cultured on hormone-free MSB_5_ medium. Values represent the mean (±) standard error of three independent experiments. Mean values followed by the different letters within a column are significantly different according to Duncan’s multiple range test (DMRT) at 5 % level

**Supplementary table 2** Effect of GA_3_, Zea, and IAA on shoot elongation of regenerated shoots from half-seed explants (derived from 1-day-old imbibed seeds) of soybean cv. DS 97–12 on SEM after 30 days of culture

| **Plant growth regulators**  **(µM)** | **Percentage of explants responding**  **(%)** | **Mean number of elongated shoots/explant** | **Mean shoot length (cm)** |
| --- | --- | --- | --- |
| Control^*^  GA_3_  0.72  1.45  2.89  4.34  5.78  ZTR  2.29  4.57  6.85  9.13  11.41  IAA  0.58  1.15  1.72  2.29  2.86 | 16.3±0.3n  60.0±0.5d  69.6±0.4a  67.3±0.6b  62.3±0.3c  54.0±0.4f  44.3±0.2j  57.3±0.5e  51.3±0.3g  46.6±0.4i  38.0±0.3k  49.6±0.4h  43.6±0.3j  38.6±0.5k  33.6±0.3l  30.3±0.3m | 1.6±0.2k  12.0±0.3c  15.3±0.5a  14.0±0.2b  12.6±0.4c  10.0±0.3d  6.6±0.3fg  10.3±0.3d  8.3±0.5e  7.3±0.6f  4.6±0.3hi  7.3±0.3f  5.6±0.4gh  4.3±0.2i  3.0±0.3j  2.3±0.4jk | 1.3±0.1m  5.2±0.2c  6.3±0.3a  5.8±0.4b  5.3±0.1c  4.7±0.1e  4.0±0.2h  5.0±0.2d  4.5±0.2f  4.2±0.1g  3.7±0.1i  3.8±0.2i  3.4±0.2j  3.1±0.1k  3.0±0.2k  2.6±0.1l |

^*^ Half-seed explants with regenerated shoots cultured on hormone-free MSB_5_ medium. Values represent the mean (±) standard error of three independent experiments. Mean values followed by the different letters within a column are significantly different according to Duncan’s multiple range test (DMRT) at 5 % level

**Supplementary table 3** Effect of IBA on rooting of elongated shoots from half-seed explants of soybean cv. DS 97–12 on RM after 30 days of culture

| **IBA**  **(µM)** | **Rooting response**  **(%)** | **Mean number of roots/shoot** | **Mean root length (cm)** |
| --- | --- | --- | --- |
| Control^*^  IBA  2.47  4.93  7.39  9.85  12.31 | 32.0±0.5e  77.6±0.3b  83.3±0.6a  78.3±0.3b  66.6±0.6c  45.3±0.3d | 2.3±0.4d  4.6±0.2bc  6.3±0.4a  5.3±0.3b  4.0±0.2c  2.6±0.1d | 2.8±0.1f  7.8±0.2c  9.6±0.2a  8.2±0.3b  6.0±0.2d  3.5±0.1e |

^*^ Elongated shoots (above 4 cm) cultured on hormone-free MSB_5_ medium. Values represent the mean (±) standard error of three independent experiments. Mean values followed by the different letters within a column are significantly different according to Duncan’s multiple range test (DMRT) at 5 % level
